# Mating Disruption for Managing the Honeydew Moth, *Cryptoblabes gnidiella* (Millière), in Mediterranean Vineyards

**DOI:** 10.3390/insects12050390

**Published:** 2021-04-28

**Authors:** Renato Ricciardi, Filippo Di Giovanni, Francesca Cosci, Edith Ladurner, Francesco Savino, Andrea Iodice, Giovanni Benelli, Andrea Lucchi

**Affiliations:** 1 Department of Agriculture, Food and Environment, University of Pisa, via del Borghetto 80, 56124 Pisa, Italy; renato_ricciardi@hotmail.it (R.R.); aphelocheirus@gmail.com (F.D.G.); francesca.cosci1@virgilio.it (F.C.); andrea.lucchi@unipi.it (A.L.); 2 CBC (Europe) srl, Biogard Division, via Zanica, 25, 24050 Grassobbio, Italy; eladurner@cbceurope.it (E.L.); fsavino@cbceurope.it (F.S.); aiodice@cbceurope.it (A.I.)

**Keywords:** chemical ecology, grapevine, Integrated Pest Management, sex pheromone

## Abstract

**Simple Summary:**

*Cryptoblabes gnidiella* has recently become one of the most feared pests in the Mediterranean grape-growing areas. Its expanding impact requires the development of effective strategies for its management. Since insecticide strategy has shown several weaknesses, we developed a pheromone-based mating disruption (MD) approach as a possible sustainable control technique for this pest. Between 2016 and 2019, field trials were carried out in two study sites in central and southern Italy, using experimental pheromone dispensers. The number of adult captures in pheromone-baited traps and the percentage of infestation recorded on ripening grapes were compared among plots treated with MD dispensers, insecticide-treated (no MD) plots, and untreated plots. Results highlighted that the application of MD may contribute to lowering the damage significantly. However, further studies aimed at clarifying the still little-known aspects of the biology and population dynamics of the honeydew moth are needed.

**Abstract:**

The demand for a reduced use of pesticides in agriculture requires the development of specific strategies for managing arthropod pests. Among eco-friendly pest control tools, pheromone-based mating disruption (MD) is promising for controlling several key insect pests of economic importance, including many lepidopteran species. In our study, we evaluated an MD approach for managing the honeydew moth (HM), *Cryptoblabes gnidiella*, an emerging threat for the grapevine in the Mediterranean basin. The trials were carried out in two study sites, located in Tuscany (central Italy, years 2017–2019) and Apulia (southern Italy, years 2016 and 2018–2019), and by applying MD dispensers only in April, in April and July, and only in July. To evaluate the effects of MD, infested bunches (%), damaged area (%) per bunch, and number of living larvae per bunch were compared among plots covered with MD dispensers, insecticide-treated plots (Apulia only), and untreated control plots. Male flights were monitored using pheromone-baited sticky traps. Except for the sampling carried out in Tuscany in 2018, where HM infestation level was very low, a significant difference was recorded between MD and control plots, both in terms of HM damage caused to ripening grapes and/or number of living larvae per bunch. Overall, our study highlighted that MD, irrespective of the application timing, significantly reduced HM damage; the levels of control achieved here were similar to those obtained with the application of insecticides (no MD). However, MD used as stand-alone strategy was not able to provide complete pest control, which may instead be pursued by growers with an IPM approach.

## 1. Introduction

In recent years, the honeydew moth (HM), *Cryptoblabes gnidiella* (Millière, 1867) (Lepidoptera, Pyralidae, Phycitinae), has become one of the most harmful moths in Mediterranean vineyards, raising concerns about the damage caused to ripening grapes [1,2,3]. Native to the Mediterranean basin, this moth is highly polyphagous, feeding on about 60 different plant species, and it has been recorded in several African and Asian countries, as well as in India, New Zealand, Hawaii, and North and South America [1,4].

Although widespread throughout the Mediterranean area, the HM has never been considered a key pest of vineyards, since it usually occurred at low density [1,3], and its impact on yield has been regarded as negligible compared to that of major vineyard pests, such as the European grapevine moth, *Lobesia botrana* (Denis & Schiffermüller) (Lepidoptera: Tortricidae), or the vine mealybug, *Planococcus ficus* (Signoret) (Hemiptera: Pseudococcidae) [5,6,7,8]. Most likely because of the increase in average temperatures, the economic impact of the HM in vineyards throughout the Mediterranean basin has been growing rapidly over the last decade [3]. In the warmer coastal vineyards of central and southern Italy, as well as in Provence (southern France), the HM has already been reported to cause severe losses in late-ripening grapes, e.g., Aglianico, Montepulciano, Sangiovese, or Grenache [3].

To date, the control of this pest has been achieved mainly through the chemical management of other key pests such as *L. botrana* [1]. For example, the use of *Bacillus thuringiensis* subsp. *kurstaki* has proven to be effective in controlling HM larval populations [1,9]. However, research focusing on the life cycle of this pest [1,2,3,4] has shown that—although the first flights are recorded in April–May—no larvae or grape damage is found before the end of July, in correspondence with the phenological phase of “majority of berries touching/beginning of ripening” (79–81 BBCH scale). Consequently, treatments against the second generation of *L. botrana* may not ensure the control of HM populations. In addition, the growing impact of this moth and the general demand for an increasingly reduced use of pesticides in agriculture require the development of specific and eco-friendly strategies for the containment of this pest.

In this context, a shift towards the development of an Integrated Pest Management (IPM) strategy for the sustainable management of the HM in vineyards is an incoming challenge for the wine industry. Among eco-friendly pest control strategies, pheromone-based mating disruption (MD) turned out to be a promising tool for the control of several insect pests [10,11,12]. Moth sexual communication is based on the release of a sex pheromone by the female, which is eventually detected by males through appropriate neurosensory structures [13,14]. The release of synthetic pheromone plumes interferes with the mate finding process [15,16,17]; thus, this technique affects the chance of reproduction of the target species, with a consequent impact on its population dynamics [18]. As mentioned above, MD has proved to be a valid control tool to manage the populations of several moths of economic importance [15,19,20,21,22,23,24] and has been successfully applied in the control of the European grapevine moth and the vine mealybug in European vineyards [5,6,8,10,25,26,27].

The aim of our study was to develop and validate a novel MD approach for managing the infestations of the HM in Italian vineyards by comparing the percentage of infested bunches, the percentage of damaged area per bunch, the number of living HM larvae per bunch, and HM trap catches among MD plots, plots treated with insecticides (hereinafter, grower’s standard), and untreated control plots. The flight pattern of *C. gnidiella* in the Mediterranean area shows four peaks [1,28,29,30,31]: the first two are in May–June and July, respectively, with relatively low captures; the third and fourth peaks are in August–October, partially overlapping and with high captures. However, although trap catches are recorded, larvae infesting bunches are hardly ever seen in May–June and appear gradually in bunches from the second half of July on [3]. For this reason, three different timings of dispenser application were tested: (*i*) dispensers applied once in April (i.e., before the beginning of the first flight); (*ii*) dispensers applied once in July (at the first appearance of larvae on bunches); and (*iii*) dispensers applied twice, once in April and once in July, with the second application aiming at reinforcing the pheromone cloud in the field before the fourth flight peak.

## 2. Materials and Methods

### 2.1. Experimental Design

Trials were carried out between 2016 and 2019, in two typical wine-growing areas: one located in Apulia (Southern Italy), in the municipality of Minervino Murge (province of Barletta-Andria-Trani), characterized by the late-ripening wine grape variety Aglianico (harvesting period: middle of October), and the other one located in Tuscany (Central Italy), in the municipality of Capalbio (province of Grosseto), on the wine grape variety Cabernet Sauvignon (harvesting period: end of September). Vineyard details are given in Table 1.

Plastic hand-applied Isonet CGX111 dispensers (Shin-Etsu Chemical Co. Ltd., Naoetsu, Japan) were tested at the application rate of 500 units/ha. The dispensers were set in a regular grid, spaced out about 4.5 m from each other. Isonet CGX111 is a reservoir pheromone dispenser consisting of two parallel brown-red polymer tubes, one filled with the HM synthetic pheromone blend (*Z*)-11-hexadecenal >27% and (*Z*)-13-octadecenal >27% and the other containing an aluminum wire that enables their placement on supports.

In Apulia, the experiment was carried out by dividing the study area into plots of 6–8 ha. The first study was conducted in 2016. Four sampling plots were established: an untreated control plot; an MD plot where Isonet CGX111 dispensers were applied only once in April (11 April); an MD plot where the dispensers were applied twice, once in April and once in July (18 July); and a plot managed according to local practices for HM control, hereinafter called the grower’s standard (treated 4 times during berry ripening with Delfin at 0.75 kg/ha, a.i. *B. thuringiensis* subsp. *kurstaki* strain SA 11). In 2018, the study was carried out by testing an MD plot with Isonet CGX111 dispensers applied in April (9 April) vs. an untreated control plot and the grower’s standard plot (treated 5 times with Delfin at 0.75 kg/ha and once with Laser 0.25 L/ha, a.i. spinosad, during fruit development/berry ripening). The trial was repeated in 2019, adding two MD plots: one with two applications, the first in April (15 April) and the second in July (22 July), both at a rate of 500 dispensers per ha, and another one where the dispensers were applied only once in July (500 per ha).

In Tuscany, the experiment was conducted by dividing the study area into plots of about 6 ha. In 2017 and 2018, the sampling was carried out testing an MD plot with Isonet CGX111 dispensers applied in April (14 April and 18 April, respectively) vs. an untreated control. In 2019, the survey was repeated, adding an MD plot where 500 dispensers per ha were applied in April (11 April) and then again in July (23 July) and another MD plot where 500 dispensers per ha were applied only in July. Experimental plots details per year are given in Table 2.

A randomized block design is not applicable to large plots required for studies on MD products (see European and Mediterranean Plant Protection Organization 2019 guidelines), and therefore each treatment (i.e., Isonet CGX111 at 500 dispensers per ha applied once in April, once in July and/or twice, grower’s standard, and untreated control) was applied to one large plot of 6–8 ha. For each treatment, 10 sampling units were selected, distributed in a grid pattern, except for the survey of 2016 in Apulia, where 6 sampling units were selected. In each of these units, 50 bunches (for a total of 300 bunches per treatment in 2016 and 500 bunches per treatment in the following years) were randomly selected and checked for HM infestation.

### 2.2. Adult Captures and Evaluation of Infestation Levels

Adult flights were monitored each year in each plot using Biogard Delta Traps (BDT sticky traps, CBC (Europe) S.r.l., Grassobbio (BG), Italy), baited with HM sex pheromone lures (NovaPher, Settimo Milanese (MI), Italy). Sticky plates and sex pheromone lures were replaced monthly; adults were counted regularly by direct observation in the field and periodically removed.

Infestation levels were evaluated on selected bunches in September, just before harvest. Three parameters were considered: percentage of HM-infested bunches, number of living larvae of *C. gnidiella* per bunch, and percentage of damaged area per bunch. The percentage of damaged area per bunch was evaluated visually as the portion of the bunch with wilted and/or damaged berries, rousers on green parts, feces, and silk.

### 2.3. Pheromone Release Rate Over the Season

In order to evaluate the gradual release of pheromone, Isonet CGX111 dispensers (*n* = 5 for sampling) during the field experiment in Apulia in 2016 were periodically collected and analyzed with GC-MS, following the method recently described by Lucchi et al. [10]. Based on internal (SEC) standard GC-MS analysis, an Agilent 6890 N (Santa Clara, CA, USA) gas chromatograph equipped with a 5973N mass spectrometer (MS) was used. MS was set as follows: EI mode, 70 eV, mass-to-charge ratio (*m*/*z*) scan between 35 and 400. HP capillary column (30 m × ID 0.25 mm × 0.25 μm film thickness, J&W Scientific, Folsom, CA, USA) with helium gas flow (1.0 mL/min) was employed for separation. The GC temperature program was 50 °C for 5 min, then increasing at 20 °C/min to 300 °C. The injector temperature was 150 °C.

### 2.4. Statistical Analysis

Estimated parameters were not normally distributed in all the plots of our samplings, and the variance was not homogeneous (Shapiro–Wilk test, goodness of fit *p* < 0.05; Levene’s test, goodness of fit *p* < 0.05). Data transformation to *ln* (x + 1) was not able to normalize the distribution or homogenize the variance. Therefore, differences between plots were tested using nonparametric statistics, i.e., the Wilcoxon test (for comparisons between two plots) or the Kruskal–Wallis test (for multiple comparisons among different plots), the latter followed by the Nemenyi post hoc test; a *p*-value of 0.05 was used as the threshold to assess significant differences. Statistical analysis was performed using R software (www.R-project.org).

## 3. Results

### 3.1. Adult Flights

**Figure 1:**
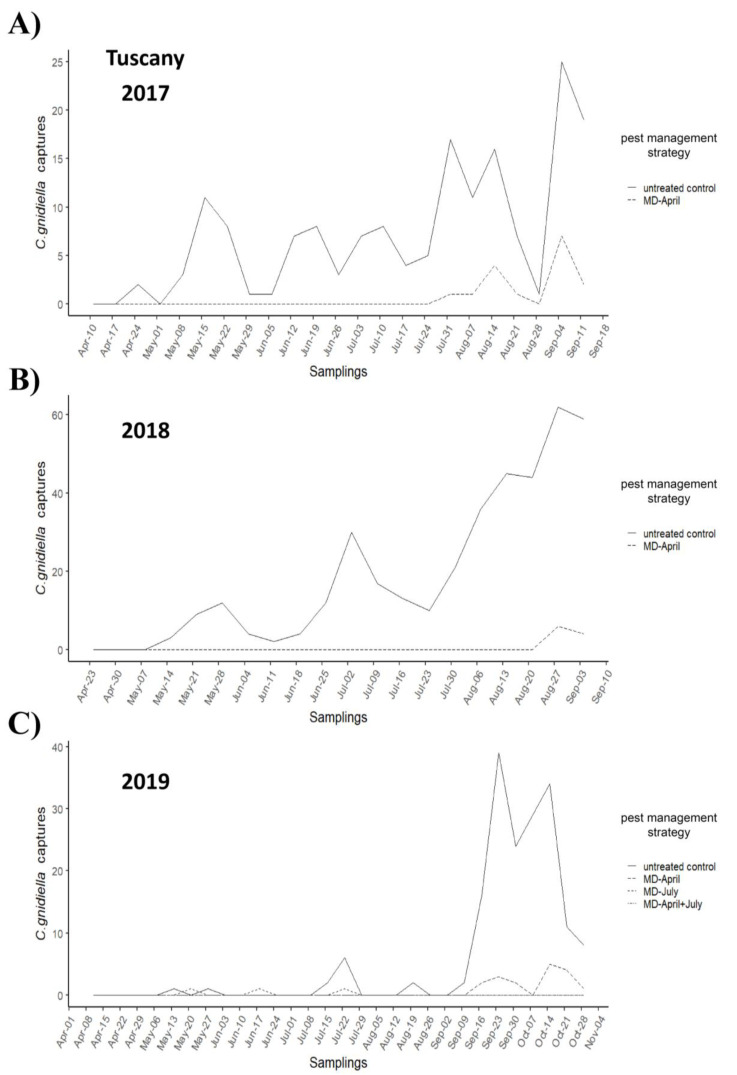
*Cryptoblabes gnidiella* flight trends in mating disruption (MD)-managed and untreated control plots in Tuscany in 2017 (**A**), 2018 (**B**), and 2019 (**C**). In all years, there is a marked contrast in the number of catches between plots under MD (dashed lines) and untreated control plots (solid line), especially from August onwards.

**Figure 2:**
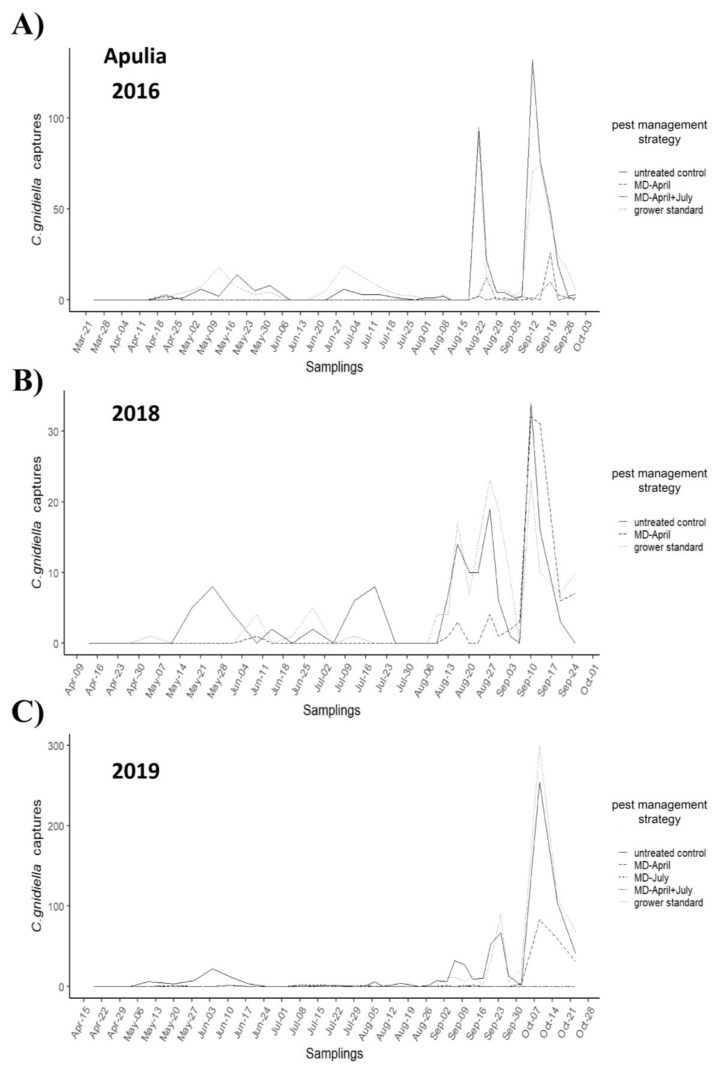
*Cryptoblabes gnidiella* flight trends in mating disruption (MD)-managed and untreated control plots in Apulia in 2016 (**A**), 2018 (**B**), and 2019 (**C**). In all years, there is a marked contrast in the number of catches between plots under MD (dashed lines), untreated control plots (solid line), and grower standard (dotted lines) especially from August onwards.

In both trials, adult flights showed a typical trend, with a first and second peak in May and July, respectively, followed by a third peak in August and a higher peak in September–October (Figure 1 and Figure 2). In Tuscany, the maximum number of male catches per trap per week occurred in the control plot during the fourth flight, with peaks of 25 adults in 2017, 62 in 2018, and 39 in 2019; MD-treated plots accounted for about 0.09% of total catches in 2017, 0.02% in 2018, and 0.10% in 2019. In Apulia, the maximum number of catches per trap per week in the untreated control plot occurred in 2016 and 2018 during the fourth flight, with peaks of 132 and 34 adults, respectively, while a maximum of 299 adults was found in 2019 in the grower’s standard plot; MD-treated plots accounted for about 0.07% of total catches in 2016, 0.22% in 2018, and 0.12% in 2019.

### 3.2. Pheromone Release Rate over the Season

**Figure 3:**
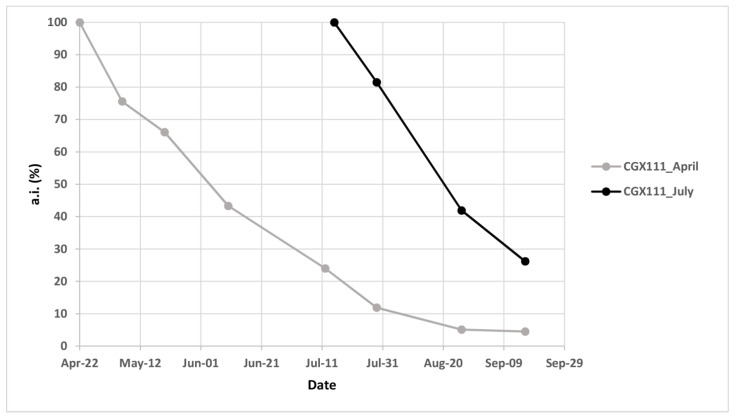
*Cryptoblabes gnidiella* pheromone load in the mating disruption dispensers applied in April or July, tested in Apulian vineyards during the 2016 growing season. a.i. = active ingredient.

Figure 3 shows the pheromone release of the Isonet CGX111 dispensers placed in the field experiment in Apulia during the 2016 grape-growing season. GC-MS results underline how the pheromone content of the dispensers decreased progressively throughout the season. During the third HM flight in August, the percentage of residual pheromone in MD dispensers applied in April was approx. 10%, and it decreased to levels below 5% during the preharvest period. Concerning MD dispensers applied in July, the residual pheromone content was about 75–80% in August, and about 25% during the preharvest period.

### 3.3. Field Experiments in Central Italy

**Figure 4:**
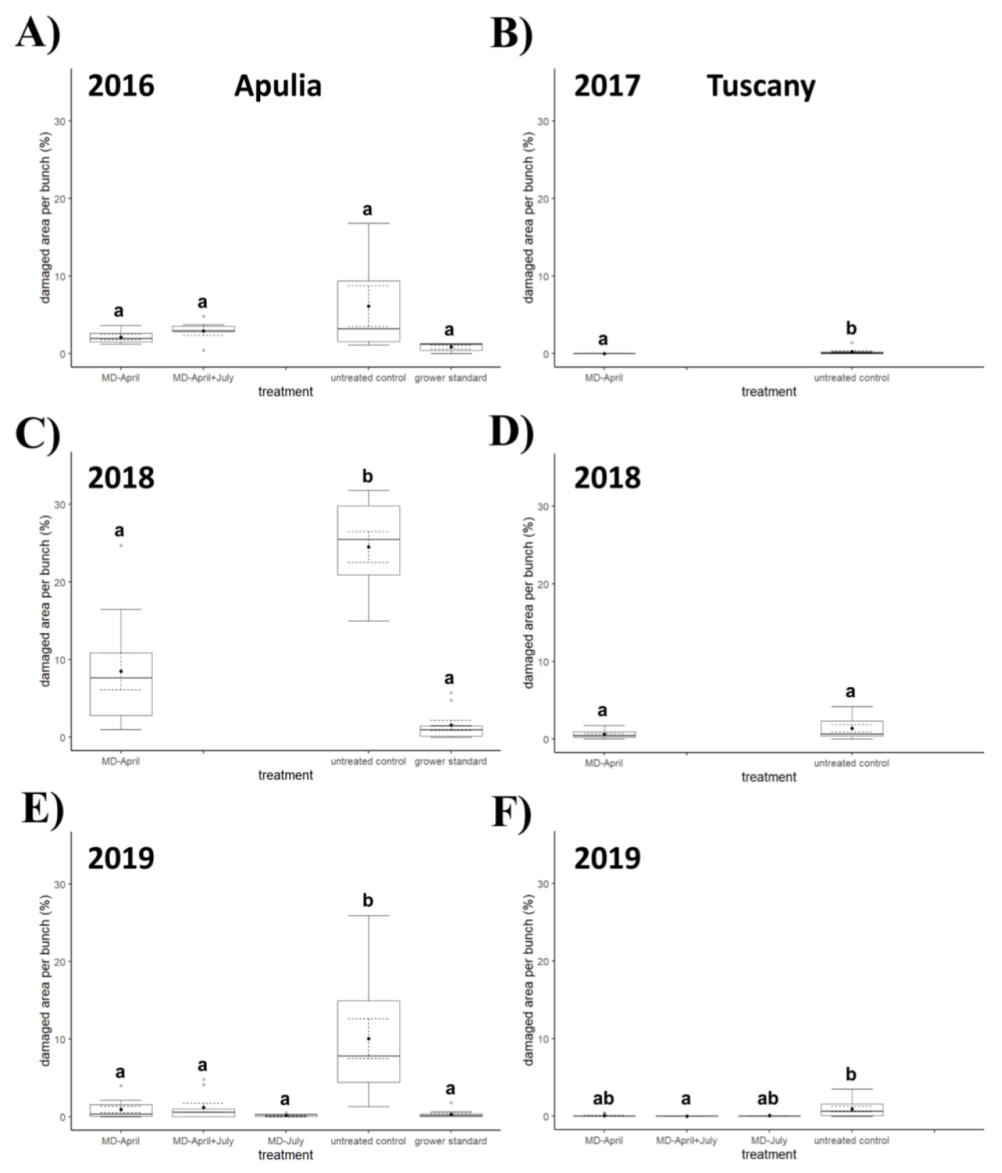
Impact of mating disruption (MD) using Isonet CGX111 dispensers on *Cryptoblabes gnidiella* damaged area (%) per bunch among treatments and untreated control plots in Apulia (**A**,**C**,**E**) and Tuscany (**B**,**D**,**F**) during three-year samplings. Boxplots indicate the median (solid line) and the range of dispersion (lower and upper quartiles and outliers) of the parameter; black dot and dashed line indicate mean value and standard error, respectively. Different letters above boxplots indicate significant differences among treatments (Wilcoxon test for comparisons between two plots or Kruskal–Wallis test for multiple comparisons, the latter followed by Nemenyi post hoc test; *p* < 0.05).

**Figure 5:**
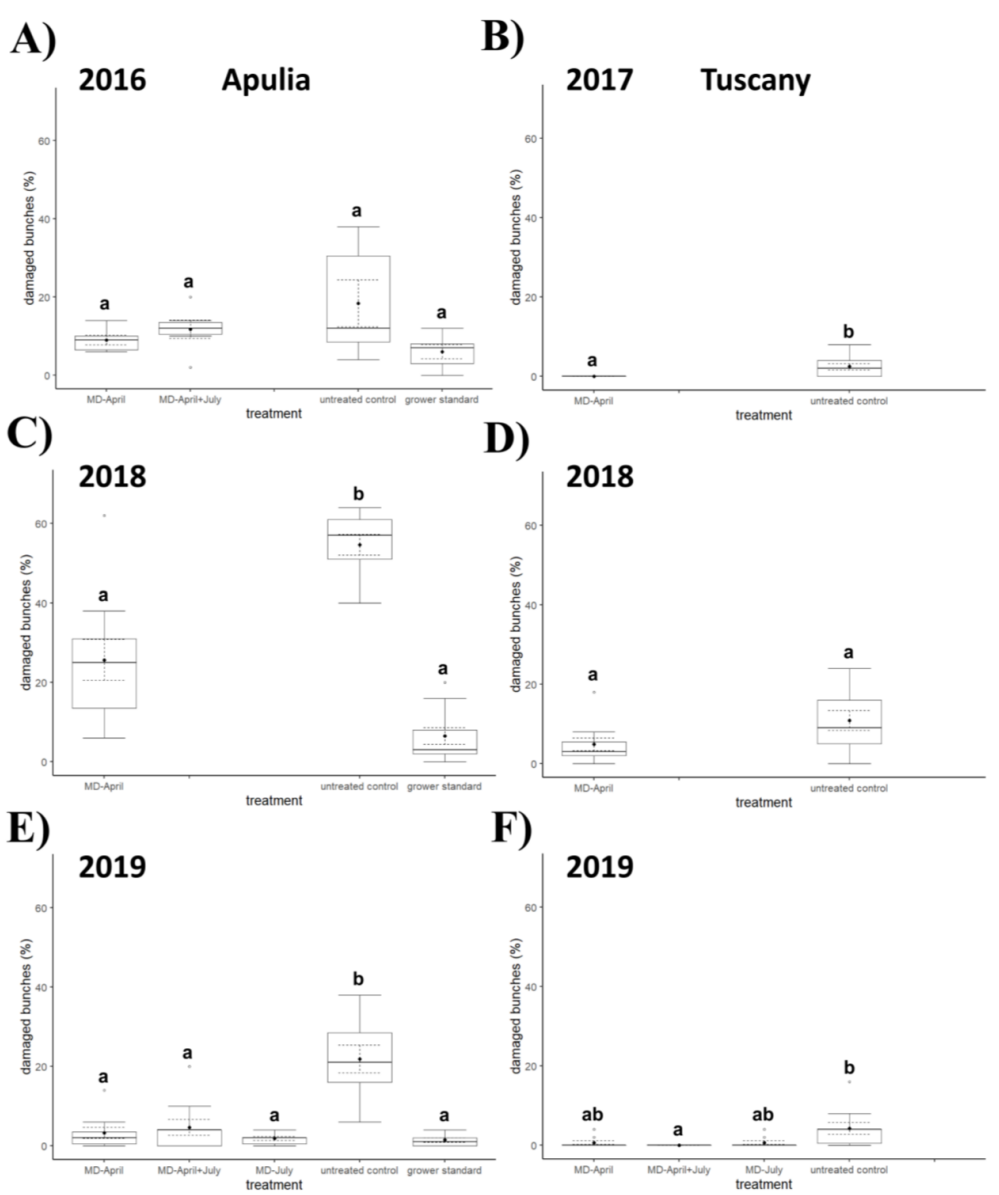
Impact of mating disruption (MD) using Isonet CGX111 dispensers on *Cryptoblabes gnidiella* infested bunches (%) among treatments and untreated control plots in Apulia (**A**,**C**,**E**) and Tuscany (**B**,**D**,**F**) during three-year samplings. Boxplots indicate the median (solid line) and the range of dispersion (lower and upper quartiles and outliers) of the parameter; black dot and dashed line indicate mean value and standard error, respectively. Different letters above boxplots indicate significant differences among treatments (Wilcoxon test for comparisons between two plots or Kruskal–Wallis test for multiple comparisons, the latter followed by Nemenyi post hoc test; *p* < 0.05).

**Figure 6:**
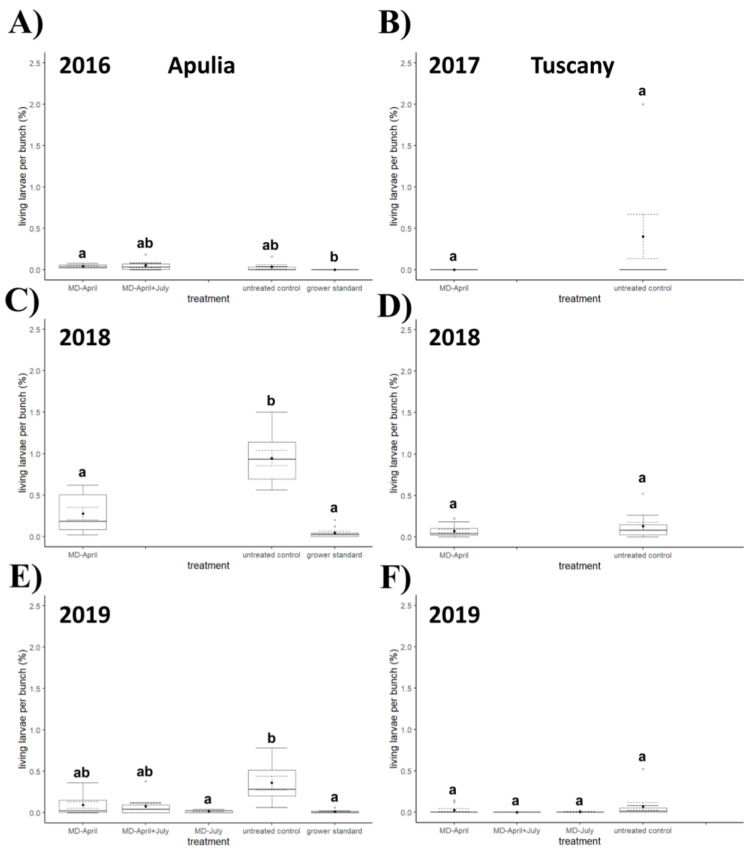
Impact of mating disruption (MD) using Isonet CGX111 dispensers on number of living larvae of *Cryptoblabes gnidiella* per bunch among treatments and untreated control plots in Apulia (**A**,**C**,**E**) and Tuscany (**B**,**D**,**F**) during three-year samplings. Boxplots indicate the median (solid line) and the range of dispersion (lower and upper quartiles and outliers) of the parameter; black dot and dashed line indicate mean value and standard error, respectively. Different letters above boxplots indicate significant differences among treatments (Wilcoxon test for comparisons between two plots or Kruskal–Wallis test for multiple comparisons, the latter followed by Nemenyi post hoc test; *p* < 0.05).

In 2017 (Figure 4, Figure 5 and Figure 6), significant differences were observed between the untreated control and the plot with dispensers applied in April in terms of percentage of infested bunches (*χ*^2^ = 7.86, *d.f.* = 1, *p* = 0.006) and percentage of damaged area per bunch (*χ*^2^ = 7.83, *d.f.* = 1, *p* = 0.006), while the difference was not significant for number of living larvae per bunch (*χ*^2^ = 2.11, *d.f.* = 1, *p* = 0.167). In 2018 (Figure 4, Figure 5 and Figure 6), no significant differences between treatments were found for any of the considered variables (percentage of infested bunches, *χ*^2^ = 3.22, *d.f.* = 1, *p* = 0.079; percentage of damaged area per bunch, *χ*^2^ = 1.76, *d.f.* = 1, *p* = 0.197; number of living larvae per bunch, *χ*^2^ = 0.58, *d.f.* = 1, *p* = 0.467). The sampling in 2019 (Figure 4, Figure 5 and Figure 6) showed significant differences among treatments in terms of percentage of infested bunches (*χ*^2^ = 14.472, *d.f.* = 3, *p* = 0.002) and percentage of damaged area per bunch (*χ*^2^ = 13.071, *d.f.* = 3, *p* = 0.004), while no significant difference emerged in terms of number of living larvae per bunch (*χ*^2^ = 7.060, *d.f.* = 3, *p* = 0.070). Both for percentage of infested bunches and percentage of damaged area per bunch, a significant difference was recorded between the untreated control and the plot with double application of dispensers in April and July (*q* = 3.976, *p* = 0.025 and *q* = 3.719, *p* = 0.042, respectively).

### 3.4. Field Experiments in Southern Italy

The Kruskal–Wallis test showed a significant difference (Figure 4, Figure 5 and Figure 6) among treatments in 2016 in terms of percentage of damaged area per bunch (*χ*^2^ = 8.130, *d.f.* = 3, *p* = 0.043) and number of living larvae per bunch (*χ*^2^ = 9.205, *d.f.* = 3, *p* = 0.027), but the Nemenyi post hoc test confirmed a significant difference only in terms of number of living larvae between the grower’s standard and the area with MD dispensers applied once in April (*q* = 3.695, *p* = 0.044). No significant differences emerged in percentage of infested bunches between treatments (*χ*^2^ = 5.284, *d.f.* = 3, *p* = 0.152).

In 2018 (Figure 4, Figure 5 and Figure 6), all the variables showed significant differences among treatments (percentage of infested bunches, *χ*^2^ = 21.690, *d.f.* = 2, *p* < 0.0001; percentage of damaged area per bunch, *χ*^2^ = 21.194, *d.f.* = 2, *p* < 0.0001; number of living larvae per bunch, *χ*^2^ = 22.041, *d.f.* = 2, *p* < 0.0001); significant differences were found for percentage of infested bunches, percentage of damaged area per bunch, and number of living larvae between the untreated control and the area with MD treatment in April (*q* = 3.341, *p* = 0.048; *q* = 3.520, *p* = 0.034; and *q* = 3.987, *p* = 0.013 respectively), and between the untreated control and the grower’s standard (*q* = 6.574, *p* < 0.0001; *q* = 6.502, *p* < 0.0001; and *q* = 6.574, *p* < 0.0001 respectively).

In 2019 (Figure 4, Figure 5 and Figure 6) all three variables again showed significant differences among treatments (percentage of infested bunches, *χ*^2^ = 24.089, *d.f.* = 4, *p* < 0.0001; percentage of damaged area per bunch, *χ*^2^ = 22.780, *d.f.* = 4, *p* = 0.0001; number of living larvae per bunch, *χ*^2^ = 23.089, *d.f.* = 4, *p* = 0.0001). In post hoc tests, significant differences were noted between the untreated control and the area with MD application in April about the percentage of infested bunches (*q* = 4.892, *p* = 0.005) and the percentage of damaged area per bunch (*q* = 4.382, *p* = 0.017); between the untreated control and the area with MD application in July, in the percentage of infested bunches (*q* = 5.434, *p* = 0.001), percentage of damaged area per bunch (*q* = 5.716, *p* = 0.0005), and number of living larvae per bunch (*q* = 5.206, *p* = 0.002); between the untreated control and the area with the application of MD dispensers in April and July about the percentage of infested bunches (*q* = 4.371, *p* = 0.017) and percentage of damaged area per bunch (*q* = 4.328, *p* = 0.019); and between the untreated control and the grower’s standard in the percentage of infested bunches (*q* = 6.020, *p* = 0.0002), percentage of damaged area per bunch (*q* = 5.694, *p* = 0.0005), and number of living larvae per bunch (*q* = 6.128, *p* = 0.0001).

## 4. Discussion

This study shows that the application of Isonet CGX111 MD dispensers contributed to reducing grape damage due to HM larval feeding (i.e., percentage of infested bunches and percentage of damaged bunch area) in most of the trials. MD has been widely recognized as an effective strategy for managing several important agricultural pests such as *Cydia pomonella* (L.) [5,24], *Grapholita molesta* (Busck) [23], *Tuta absoluta* (Meyrick) [22], *Eupoecilia ambiguella* (Hübner), and *L. botrana* [6,26,27,32], as well as mealybugs such as *Planococcus ficus* (Signoret) and scale insects such as *Aonidiella aurantii* (Maskell) [8,33]. Concerning *C. gnidiella*, pheromone-baited traps have been used to track flights of this moth in vineyards, orchards, and uncultivated areas [3], but little has been done aiming at the application of MD for the control of this moth [2,34,35].

In our experiments, differences in bunch damage between MD treated and untreated control plots failed to show significance in Apulia in 2016 and in Tuscany in 2018, but it must be pointed out that a high variability among sampling units within the untreated control plot was recorded in these trials. The irregular distribution of the damage is a consequence of the irregular distribution of larvae, which is likely related to the vigor of the plants and the concomitant presence of other pests [1,2,3,4]. As far as the number of living larvae per bunch is concerned, it was generally higher in control plots than in MD plots in Apulia, while differences were never significant in Tuscany; the number of living larvae found was too low in all study years to be considered conclusive.

In all trials, a considerable reduction was observed in the number of trap catches in MD-treated plots compared to control plots. When the dispenser application was performed also or only in July, an almost complete trap catch shut-down was achieved. However, although pheromone-baited traps can provide useful information about the efficacy of MD products [36], crop damage reduction was less pronounced than trap catch reduction. These findings confirm what is stated in the official EPPO guidelines for efficacy evaluation of mating disruption products. HM catches in the monitoring traps should not be considered as the only indicator for MD efficacy assessment [36,37,38].

No substantial differences emerged in terms of percentage of infested bunches among MD-treated plots. In all the trials, no significant differences in bunch damage emerged between the plots treated with the application of MD dispensers in April, the application of MD dispensers in July, or the double application of MD dispensers in April and July. However, as indicated by the pheromone release curves and the trap catches, the application of MD dispensers in April was not sufficient to provide an adequate pheromone release during the preharvest period. The level of residual pheromone in August–October may not be sufficient to provide adequate HM control for late-ripening varieties. Furthermore, as already mentioned above, when MD dispensers were applied in July, a considerable reduction in adult catches during the fourth flight was observed in comparison to the untreated control, the grower’s standard, and the plot where MD was applied in April. It may therefore be assumed that by placing the MD dispensers in July, MD efficacy could be extended up to harvest time also on very late ripening varieties.

In Apulia, the efficacy of MD in bunch damage reduction was always comparable to that of the grower’s standard treatments. The level of HM damage reduction achieved both with MD alone and with insecticide applications only (no MD) was not complete and was considered not acceptable by growers. However, strategies consisting in the combined use of MD and a reduced number of insecticidal applications (e.g., *B. thuringiensis*) are being successfully used for the control of many lepidopteran pests, and they may constitute a valuable tool for HM control as well.

It is known that insecticide treatments focused on controlling the third-generation eggs and young larvae of *L. botrana* in late July/beginning of August may have an effect against *C. gnidiella*. However, as the life cycles of the two pests are not synchronous, insecticide treatments against the HM must be extended to the end of August or, in the case of late-ripening varieties, all through September.

As the HM is commonly present alongside *L. botrana*, a further strategy could be represented by the application of double dispensers for the control of both pests, followed by insecticide treatments as needed. Although the life cycles of the two species are not strictly synchronous, the gradual release of pheromone provided by the dispensers can cover the period of activity of both species. In a first trial conducted in Israel with MD against *L. botrana* and *C. gnidiella*, no significant differences were observed between plots treated against one vs. two pests. Moreover, in plots treated with pheromones against both pests, no pesticides were applied [2]. However, in some cases where MD was applied against more than a single target pest, mixed results were obtained [2]. In addition, it should be pointed out that the efficacy of MD in the control of both *L. botrana* and the HM has not been verified yet at high population densities of the latter [2].

Further studies aimed at clarifying the still poorly explored aspects of HM biology are needed. It is unclear whether the high number of catches from August onwards are adults, which have completed the life cycle (egg to adult) in the vineyard or are adults migrating from the surrounding environment. Moreover, the life cycle of the HM may be influenced by several factors such as temperature and relative humidity [39,40], causing wide fluctuations in HM populations from year to year. Shedding light on the issues concerning the ecology of this moth in the field and the factors influencing the growth of populations in the vineyard are crucial for calibrating the use of MD and its further development.

## 5. Conclusions

This study shows that MD may represent an effective and sustainable method for the control of the HM in vineyards. However, at least with the formulations used in our trials, MD was not able to provide complete pest control as a stand-alone strategy, and it may need to be supported by additional insecticide treatments to reduce the population of this pest to a level, which could be considered acceptable by growers. Moreover, the HM is a highly polyphagous species, which likely spends part of its life cycle on wild plants and then appears in the vineyards at the beginning of grape ripening, most probably attracted by volatile compounds [1]. One of the caveats for determining a pest’s susceptibility to pheromone MD is the prevalence of migrating females, as MD cannot provide protection against mated females coming from outside the area treated with the disruptant [15]. For this reason, further studies on the HM phenology are necessary, especially in the months of May and June. In addition, when high populations occur, MD should be regarded as a control method within an IPM strategy [15,17,41]. An increase in knowledge about HM occurrence on wild host plants and abiotic factors influencing its population dynamics is crucial to enhance the application of MD against this emerging threat to Mediterranean vineyards.

## Figures and Tables

**Table 1:** Sites and crop details of the vineyards where Isonet CGX111 dispensers were tested to manage *Cryptoblabes gnidiella* populations.

Site	Variety	Harvesting Period	Row Spacing (m)	Space within Rows (m)
Tuscany (Capalbio) 42°25′51.78″ N 11°25′4.82″ E	Cabernet Sauvignon	End of September	1.3	0.6
Apulia (Minervino Murge) 41°8′56.72″ N 16°2′16.09″ E	Aglianico	Middle of October	2.3	0.8

**Table 2:** Treatments tested in the different years in Minervino Murge and Capalbio vineyards. A: Isonet CGX111 dispensers applied only in April; AJ: Isonet CGX111 dispensers applied twice, once in April and once in July; J: Isonet CGX111 dispensers applied only in July; C: untreated control; GS: grower’s standard (Delfin at 0.75 kg/ha, a.i. *Bacillus thuringiensis kurstaki*—SA 11 and Laser 0.25 L/ha, a.i. spinosad); ‘-’: treatment not included in the study.

Year	Site	Treatements				
2016	Minervino Murge	A	AJ	-	C	GS
2017	Capalbio	A	-	-	C	-
2018	Minervino Murge	A	-	-	C	GS
	Capalbio	A	-	-	C	-
2019	Minervino Murge	A	AJ	J	C	GS
	Capalbio	A	AJ	J	C	-

## Data Availability

Data are contained within the article.
